# Endocrine Disruptors and Obesity

**DOI:** 10.1007/s13679-017-0240-4

**Published:** 2017-02-15

**Authors:** Philippa D. Darbre

**Affiliations:** School of Biological Sciences, University of Reading, Reading, RG6 6UB UK

**Keywords:** Adipogenesis, Bisphenol A, Diethylstilbestrol, Endocrine disruptor, Endocrine-disrupting chemicals, Obesity, Obesogen, Paraben, Peroxisome proliferator-activated receptor, Persistent organic pollutants, Tributyltin

## Abstract

**Purpose of Review:**

The purpose of this review was to summarise current evidence that some environmental chemicals may be able to interfere in the endocrine regulation of energy metabolism and adipose tissue structure.

**Recent Findings:**

Recent findings demonstrate that such endocrine-disrupting chemicals, termed “obesogens”, can promote adipogenesis and cause weight gain. This includes compounds to which the human population is exposed in daily life through their use in pesticides/herbicides, industrial and household products, plastics, detergents, flame retardants and as ingredients in personal care products. Animal models and epidemiological studies have shown that an especially sensitive time for exposure is in utero or the neonatal period.

**Summary:**

In summarising the actions of obesogens, it is noteworthy that as their structures are mainly lipophilic, their ability to increase fat deposition has the added consequence of increasing the capacity for their own retention. This has the potential for a vicious spiral not only of increasing obesity but also increasing the retention of other lipophilic pollutant chemicals with an even broader range of adverse actions. This might offer an explanation as to why obesity is an underlying risk factor for so many diseases including cancer.

## Introduction

The endocrine system plays a fundamental role in regulating the metabolism of fats, carbohydrates and proteins and in ensuring that these fuels provide for the energy needs of the body at all times. Hormones are responsible for storage of excess fuel in times of plenty and mobilisation of fuel in times of need, and most notably in maintaining constant levels of blood glucose. Any alteration to these hormonally driven processes can be expected to lead to an imbalance in metabolism. The main store of energy in the body is provided by fat held in adipocytes in the adipose tissue, and it is now recognised that the adipose tissue is also under endocrine control and can itself act as an endocrine organ capable of secreting hormones [1]. Interference in hormonal control of adipose tissue functions can therefore also lead to inappropriate deposits of fat and, hence, obesity.

Over recent years, many environmental chemicals have been shown to disrupt the actions of hormones and have been termed endocrine-disrupting chemicals (EDCs) or endocrine disruptors [2•]. Although much of the research has focused on disruption of reproduction through interference with steroid hormone actions and on disruption to thyroid hormone action [2•], there are increasing reports that some EDCs can also interfere with regulatory processes in metabolism and in the control of adipocyte function, resulting in imbalances in the regulation of body weight, which can lead to obesity [3•, 4•, 5•]. Such chemicals have been termed “obesogens” [6, 7•]. Increase in obesity, defined as a body mass index of over 30 kg/m^2^, has become a global problem over recent decades. Over 20% of adults are now obese in the UK and over 30% of adults are obese in the USA [8•]. Furthermore, obesity in children is also increasing in westernised countries, and in the USA, around 20% of children aged 3–17 years are obese [8•]. Although there are genetic determinants which lead to inherited predisposition, and there are environmental influences from excessive food intake combined with lack of exercise in modern life, these alone cannot account for the current disease trends. This review will present evidence that EDCs may contribute to obesity through interfering with the control of energy metabolism and adipose tissue regulation, causing an altered balance towards weight gain and obesity, despite normal diet and exercise patterns.

## Sources of Endocrine Disruptors

**Fig. 1:**
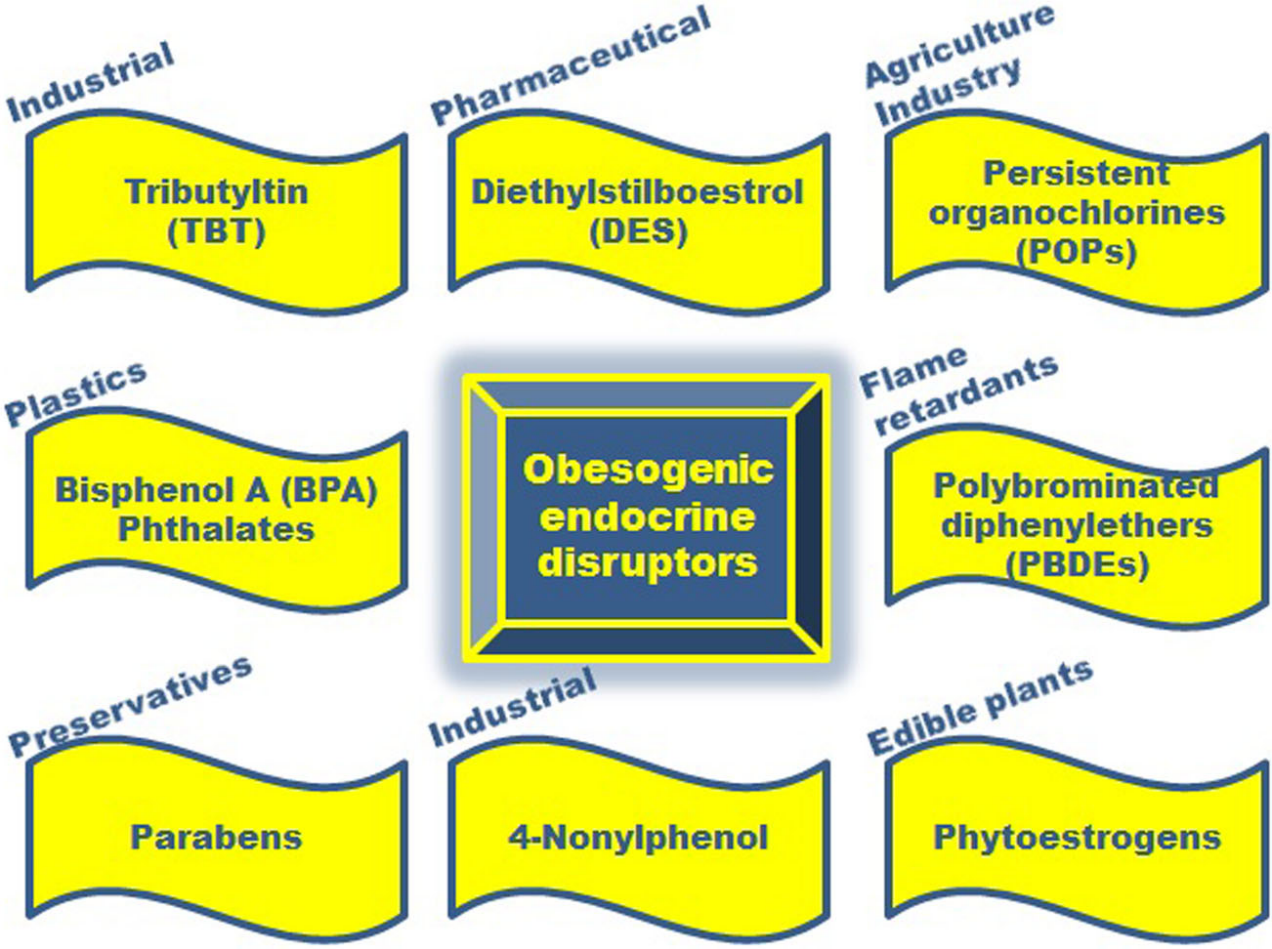
Environmental endocrine disruptors which have been shown to possess obesogenic properties, as discussed in this review

An endocrine disruptor has been defined as “an exogenous substance that causes adverse health effects in an intact organism, and/or its progeny, consequent to changes in endocrine function” [9]. Some of these compounds are present in nature (e.g. plant phytoestrogens), but the majority are synthetic chemicals which have been released by human activities into the environment without any prior knowledge of their effects on ecosystems or human health. The human population is now ubiquitously exposed to such chemicals in daily life, in indoor as well as outdoor environments, through their use in pesticides/herbicides, industrial and household products, plastics, detergents, flame retardants and as ingredients of personal care products [2•]. Intake to the human body may be oral, inhalation or dermal absorption [2•]. Figure 1 indicates some of the subset of endocrine disruptors which have been shown to possess obesogenic properties: their origins and endocrine-disrupting activities are outlined below, and the remainder of the review will discuss the evidence that they possess also obesogenic properties.

- *Tributyltin* (TBT) is an environmental contaminant from its use as a biocide in antifouling paints applied to the hulls of ships, and it has been reported to cause imposex in molluscs and to masculinise female fish [10]. TBT can inhibit aromatase, which is the enzyme responsible for the conversion of testosterone into estrogens [11, 12].
- *Diethylstilbestrol* (DES) is a synthetic non-steroidal oestrogen that was first synthesised in 1938 [13] and then prescribed to several million women between 1940 and 1971 to prevent threatened miscarriage in the first trimester [14], before untoward side effects stopped further prescription [15]. It has also been used to enhance fertility in farm animals used for meat supply [15].
- *Persistent organic pollutants* (POPs) are stable man-made compounds that do not readily degrade and tend to persist in the environment and bioaccumulate [2•]. Many are lipophilic and therefore become stored in fatty tissues, passing up the food chain in animal fat. From use as an insecticide, both dichlorodiphenyltrichloroethane (DDT) and its breakdown product dichlorodiphenyldichloroethylene (DDE) remain widely present in human adipose tissue [16] and are endocrine disrupters [2•]. Polychlorinated biphenyls (PCBs) are industrial POPs which are also widely measurable in human adipose tissue [17] and have been shown to be endocrine disrupters [2•].
- *Bisphenol A* and *phthalates* are used in the manufacture of plastics. Bisphenol A (BPA) is used for its cross-linking properties in the manufacture of polycarbonate plastics and epoxy resins, which are now ubiquitous in consumer products such as water bottles, linings of water pipes, coatings on food and beverage cans, thermal paper and dental sealants. It is listed as a high production volume chemical by the Organisation for Economic Cooperation and Development (OECD) [18]. It can leach out from plastic containers [19] and has endocrine-disrupting properties [20, 21]. Phthalates are esters of phthalic acid and are used mainly as plasticizers to increase the flexibility, transparency and durability of plastic materials. They are found in many consumer products including adhesives, paints, packaging, children’s toys, electronics, flooring, medical equipment, personal care products, air fresheners, food products, pharmaceuticals and textiles. Many of the phthalates are also listed by the OECD in their 2004 list of high production volume chemicals [18] and possess endocrine-disrupting properties [22, 23].
- *Polybrominated diphenyl ethers* (PBDEs) and *polybrominated biphenyls* are widely used as flame retardants. They are now detectable in human tissues [24] and have endocrine-disrupting properties through interference in thyroid function [25].
- *4*-*Nonylphenol* is one of the long-chain alkyl phenols used as a surfactant in industrial and domestic applications worldwide which is listed as a high production chemical by the OECD [18] and is an endocrine disrupter due to its estrogenic activity [26].
- *Parabens* (alkyl esters of *p*-hydroxybenzoic acid) are used as antimicrobial agents for the preservation of personal care products, foods, pharmaceutical products and paper products. They are widely present in human tissues including breast tissue and have estrogenic properties [27].
- *Phytoestrogens* are produced naturally by plants and as such are ingested by humans in the diet in edible plant material. Isoflavones such as genistein and daidzein are found in soybeans, legumes, lentils and chickpeas. Phytoestrogens are so named for their estrogenic activity [28]. On the general assumption that naturally occurring compounds are more beneficial than synthetic compounds, phytoestrogens have been embraced much more positively by society than the synthetic xenoestrogens, and as such, potential benefits tend to have been overemphasised compared with adverse effects, such as those potentially related to obesity.

## Developmental Susceptibility to Obesogenic EDCs

**Fig. 2:**
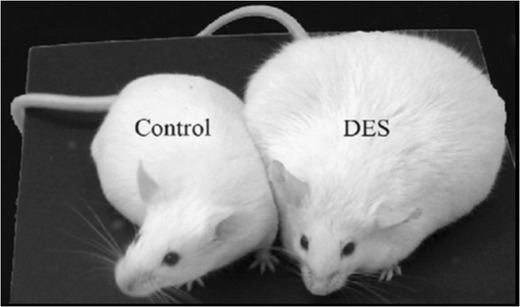
Exposure to diethylstilbestrol (*DES*) during the neonatal period predisposes to obesity in mice at 4–6 months of age. Reproduced from Newbold et al. [99], with permission from Elsevier

An especially sensitive time frame for exposure to obesogens has been found to be either prior to birth in utero or in the neonatal period [29]. Animal models have shown that exposure of pregnant mice to TBT results in offspring which are heavier than those not exposed [30]. Neonatal mice exposed to the synthetic oestrogen DES have also been reported to have increased body weight [31]. Figure 2 shows a representative photomicrograph at 4–6 months of age of control and neonatal DES-treated female mice: the mice were treated on days 1–5 of age with 1 μgDES/kg body weight/day, and obesity was evident by 4–6 months of age [31]. This serves to demonstrate the obesogenic consequences of exposure to a potent oestrogen at an inappropriate developmental stage. Other animal models have shown that exposure to some PCB congeners [32], to BPA [33], to bisphenol S, an increasingly used substitute for BPA [34•], and to a commercial mixture of PBDEs [35] can also predispose animals to weight gain. Post-weaning exposure of C57BL/6J mice to methylparaben by oral gavage also increased adiposity [36•], but such effects were not observed with the longer alkyl chain butylparaben [36•]. This is in marked contrast to the positive association observed for parabens between oestrogenic activity and longer alkyl chains [27].

The reported rise in obesity of children under 2 years of age [37, 38] is also suggestive of alterations during development. It would seem unlikely that such young children would have been affected by eating so much more food and taking so much less exercise than in previous generations, and current explanations revolve around an altered environment in utero or postnatally which is affecting fat deposition in early life [4•]. This is backed by epidemiological evidence [39–41]. Studies of babies born to mothers who smoked tobacco during pregnancy have been found to have a low birth weight, but paradoxically to then be at increased risk of obesity [42], and meta-analysis of multiple studies confirms that early-life exposure to some components of tobacco smoke can lead to later obesity [43]. A study of children in the Faroe Islands has shown that prenatal exposure to PCBs and DDE contained in dietary seafood is also associated with increased body weight [44•]. Interestingly, the effects in this study showed a non-monotonic response with the lower doses causing a greater weight gain, an effect which is consistent with many other actions of the EDCs where non-monotonic responses are commonly observed [45]. Other studies have shown that early-life exposures to some POPs [46] and bisphenol A [47] are also associated with increased body weight in young children.

Some studies suggest that such effects may even be inherited through future generations without the need for further exposure. Transgenerational studies have shown that pregnant mice exposed to TBT produce offspring with greater fat deposits, and this phenotype was found to be passed on to the F3 generation, although there was no further TBT exposure [48•]. Other heritable traits towards obesity in rodents have been demonstrated following exposure to BPA and phthalates (diethylhexyl and dibutyl) [49•] and DDT [50•].

## Mechanisms of Action of Obesogenic EDCs

**Fig. 3:**
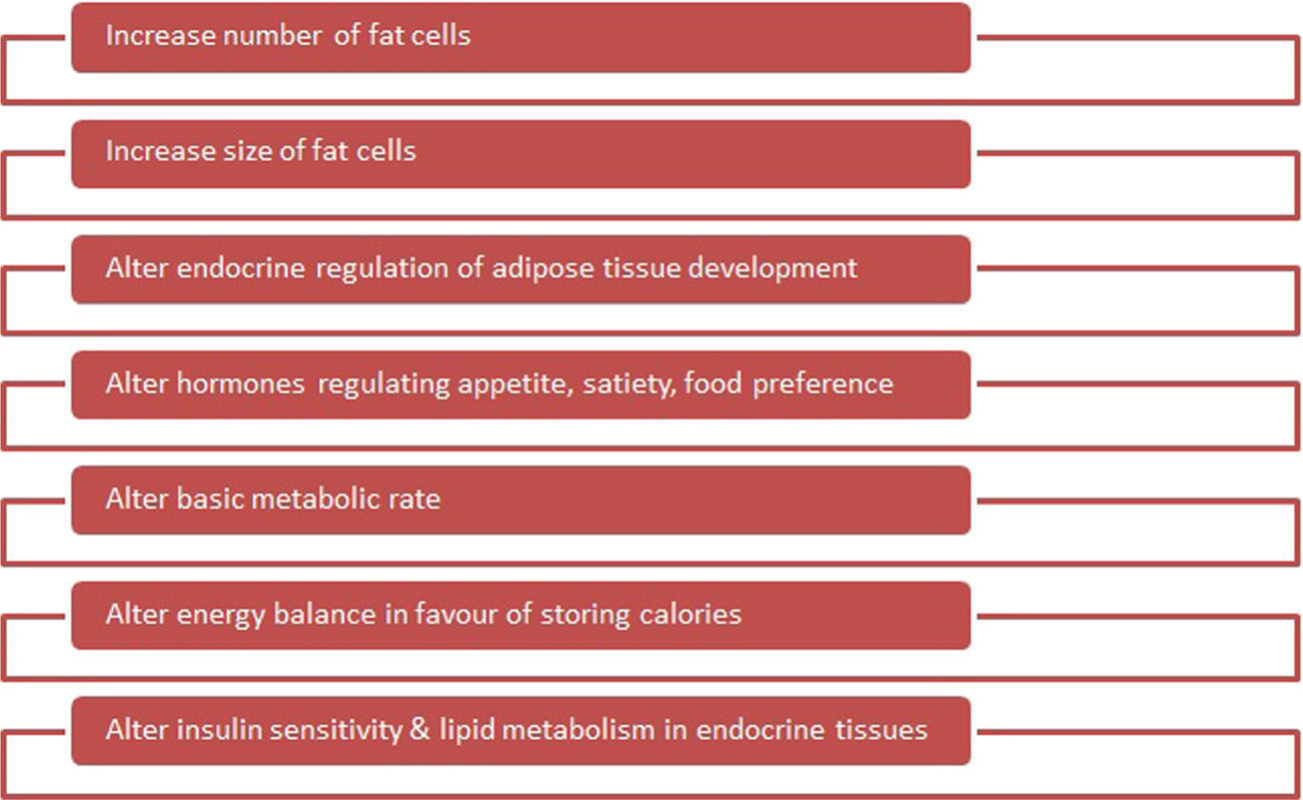
Summary of the mechanisms of action of obesogenic endocrine disruptors

Obesogens cause weight gain by altering lipid homeostasis to promote adipogenesis and lipid accumulation, and this may occur through multiple mechanisms, outlined in Fig. 3. This may occur through increasing the number of adipocytes, increasing the size of adipocytes or altering the endocrine pathways responsible for the control of adipose tissue development. In general, early developmental changes (in utero or postnatally) involve an increase in adipocyte numbers, whilst changes later in life during adulthood tend to involve mainly an increase in the size of adipocytes [51]. Current evidence suggests that adipocyte numbers are set by the end of childhood and that any increase in adipocyte numbers in early life tends to be permanent [51]. This has profound consequences for the timing of exposure to obesogens, implying that alterations during early life would be passed on into adulthood, which would not be reversed [4•]. Other mechanisms of interference by obesogens may involve altering hormones that regulate appetite, satiety and food preferences, altering the basal metabolic rate or altering the energy balance to favour storage of calories. Finally, mechanisms may involve alterations to insulin sensitivity and lipid metabolism in endocrine tissues such as the pancreas, adipose tissue, liver, gastrointestinal tract, brain or muscle.

At a molecular level, obesogens can act by interfering with nuclear transcriptional regulators that control lipid flux and/or adipocyte proliferation/differentiation, such as the peroxisome proliferator-activated receptors (PPARα, PPAR-δ and PPAR-γ) and steroid hormone receptors. These receptors all function as ligand-activated transcription factors in that, after binding ligands, they are enabled to bind to response elements in the DNA and act to regulate specific patterns of gene expression [2•]. EDCs which can bind to these nuclear receptors have the potential to cause inappropriate responses, including obesity.

### Obesogens Acting through Interference with PPARs

PPARs can bind a wide range of unsaturated fatty acids and therefore act as lipid sensors to regulate lipid homeostasis. PPARγ functions in energy storage through its regulatory actions on adipogenesis and has therefore been defined as a master regulator of fat cell development [52]. PPARs act by heterodimerisation with retinoid X receptors (RXRs), and activation of RXR-PPARγ favours the differentiation of adipocyte progenitors and preadipocytes in adipose tissue and regulates lipid biosynthesis and storage [53]. In contrast, activation of the RXR-PPARα heterodimer stimulates β-oxidation breakdown of fatty acids [54]. Several EDCs have now been shown to alter adipogenesis through interfering in PPARγ actions [55].

Both tributyltin and triphenyltin are nanomolar affinity ligands for the RXR-PPARγ heterodimer [30, 56] and stimulate 3T3-L1 preadipocytes to differentiate into adipocytes [30, 56, 57] in a PPARγ-dependent manner [58, 59]. Using the same 3T3L1 cell model, BPA [60], nonylphenol [61] and the fungicide triflumizole [62] have also been shown to promote adipogenesis. The phthalate metabolite mono(2-ethyl-hexyl)phthalate is a known potent and selective activator of PPARγ [63] that promotes the differentiation of 3T3-L1 cells into adipocytes [64]. Although many phthalates show greater responses through PPARα than on PPARγ [65], it may be the metabolites which act through PPARγ that cause weight gain. Phthalate metabolites in excess of several micrograms per litre have been measured in over three quarters of urine samples from the US population [66], and epidemiological studies have noted an association between phthalate metabolites and increased waist circumference [67, 68]. More recent work has reported that benzyl butyl phthalate can promote adipogenesis in 3T3-L1 preadipocytes [69], and so there may be some variation between different esters.

Parabens have also been shown to promote adipocyte differentiation in 3T3-L1 cells [70]. Adipogenic potency increased with the linear length of the alkyl chain [70] and was associated with PPARγ activation [71]. It now appears likely that any ligand which can bind to the PPARγ will be able to influence adipogenesis and obesity [71]. Since so many of such ligands are present in human adipose tissue, it is probable that they may also be able to act in combination through additive mechanisms. This may then enable mixtures of such ligands to stimulate adipogenesis at lower concentrations than needed for each alone. Such additive effects are recognised for other endocrine-disrupting mechanisms, most notably in the context of combinations of oestrogenic compounds acting via oestrogen receptors to increase proliferation of oestrogen-responsive cells [2•].

### Obesogens Acting through Interference with Steroid Receptors

Steroid hormones can influence lipid storage and fat deposition and can therefore also impact on adipose tissue development [72]. Hormone replacement therapy based on oestrogens can protect against many age-related changes in adipose depot remodelling at menopause [73]. Dietary soy phytoestrogens, such as genistein and daidzein, modulate oestrogen receptor signalling and reverse truncal fat accumulation in postmenopausal women [74], an effect which has been demonstrated also in ovariectomized rodent models [75]. However, foetal or neonatal oestrogen exposure can have the opposite effect and lead to obesity later in life, which emphasises, again, that timing of exposure can be important in the consideration of outcomes. Rodents treated with phytoestrogens during pregnancy or lactation were observed to develop obesity at puberty [76], especially in males [77], which might question the wisdom of consumption of soy-based products by baby boys. Neonatal exposure of female mice to the synthetic oestrogen DES initially led to depressed body weight, but was followed by long-term weight gain in adulthood [78, 79], although notably not in male mice [80], demonstrating the obesogenic effect of a potent oestrogen on adipogenesis which can be gender-specific.

Whilst some EDCs may act directly through cellular steroid receptors, other EDCs may act less directly by stimulating oestrogen synthesis. Adipose tissue is known to be a site of oestrogen synthesis, and the cytoplasm of adipocytes contains the cytochrome P450 enzyme aromatase which converts testosterone to oestrogens. Several EDCs are now known to be able to influence intracellular aromatase activity [81] and could therefore act indirectly to raise the intracellular levels of oestrogen in adipocytes with a consequent increase in obesity not only in women but also in men [82].

### Obesogens Act through Aryl Hydrocarbon Receptors

The aryl hydrocarbon receptor (AhR) is a ligand-activated transcription factor that senses the presence of foreign compounds such as POPs and leads to the activation of cytochrome P450 enzymes needed for their clearance from the body [2•]. However, AhR can indirectly influence adipogenesis through altering PPARγ expression, and some POPs with obesogenic activity, which can be inhibited by AhR antagonists, have been suggested to function through this mechanism of action [32].

### Obesogens Act by Altering Recruitment of Fat Cells

Mature adipocytes are generated from multipotent stromal cells (MSCs) of foetal and adult tissues [83]. These MSCs can differentiate into several different cell types in vitro, including not only adipose tissue but also bone, cartilage and muscle, and regulation of these processes is essential for homeostasis. Exposure of pregnant mice to TBT has been shown to result in MSCs which differentiate in offspring preferentially into adipocytes rather than bone and, furthermore, which have epigenetic alterations in the methylation status of some adipogenic genes [58]. Similar results were obtained following prenatal exposure to the fungicide triflumizole [62], which demonstrates that some EDCs can act by altering recruitment as well as differentiation of fat cells. A sensitive time for such alterations would be during the development of adipose tissue in early life.

### Obesogens act by altering appetite, satiety and food preferences

Another mechanism of EDC action may be through altering the energy balance between energy intake and energy expenditure, especially by altering appetite, satiety and food preferences. Although BPA has been shown to induce obesity in experimental studies [33] and is present in over 90% of urine samples in the US population [84], no direct association has been established between serum BPA levels and amount of body fat [85]. However, more recently, BPA levels have been found to correlate in humans with circulating levels of leptin and ghrelin [86•]. These are hormones secreted by the adipose tissue which act in opposing ways to regulate hunger, with leptin being inhibitory and ghrelin being stimulatory. Alterations to the circulating levels of these hormones suggest that BPA may be able to act by interfering with hormonal control of hunger and satiety [86•], which is corroborated by a recent report of increased production of leptin mRNA in a 3T3L1 adipocyte model after 3 weeks of exposure to 1 nM BPA [87]. In a mouse model, post-weaning exposure to methylparaben also increased serum leptin levels [36•], which begs the question as to whether any association between paraben exposure and leptin levels might be observed in human studies.

## The Vicious Spiral of Obesogenic EDC Actions and Human Health

**Fig. 4:**
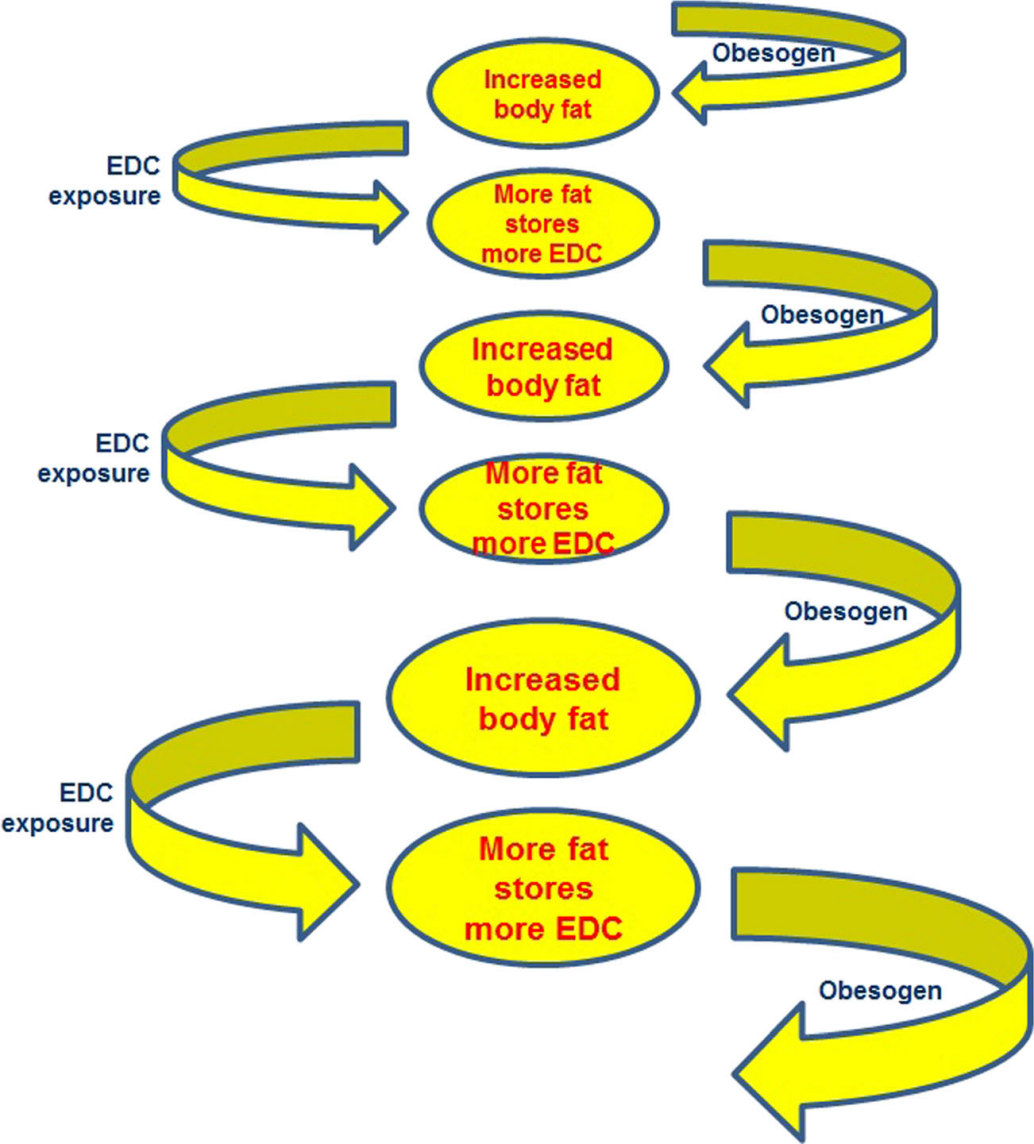
The “vicious spiral” of obesogenic activity and lipophilic properties of endocrine disruptors. The obesogenic activity of endocrine-disrupting chemicals (*EDCs*) results in increased body fat: since EDCs are lipophilic, more EDCs will be stored as the amount of body fat increases. This may cause an upwards spiral towards increasing body fat and, therefore, increasing body burden of EDCs, and indeed of other lipophilic environmental pollutant chemicals as well

Obesity is not just a matter of carrying excess weight through life, but has been established as an underlying risk factor for many diseases including metabolic syndrome [88], diabetes [89], cardiovascular disease [90] and cancer [91]. Coincidentally, development of many of these diseases has also been linked to exposure to EDCs [2•]. In this context, it is noteworthy that many EDCs are lipophilic, and POPs in particular are known to bioaccumulate in body fat over the years. It is therefore plausible that the link of obesity to disease relates not just simply to the deposition of fat but to the increased retention of lipophilic pollutants in the greater volume of fat. Figure 4 illustrates the potential for a “vicious spiral” to be set up whereby obesogens act to increase the amount of fat stored (be it by increased cell volume or increased cell number), which would be followed by greater retention of lipophilic obesogens and which would then lead onward in an increasing spiral of greater body fat and even more lipophilic pollutant retention. In this sense, obesogens may be self-fulfilling and be able to increase capacity for their own retention.

Although such a mechanism may enable obesity to be associated with a greater body burden of obesogens, there are also many other lipophilic pollutant chemicals which have adverse effects on the human body other than through their obesogenic activity. Therefore, the obesogenic nature of some EDCs may enable increased storage of a wide range of other lipophilic environmental pollutants with adverse properties, including non-obesogenic EDCs and non-obesogenic non-EDC environmental chemicals. Retention of such a complex mixture of pollutant chemicals offers the potential for additive or complementary mechanisms to aggravate disease processes. For example, in considering the hallmarks of cancer [92], whilst sustained proliferation of endocrine-regulated cancers may be driven by EDCs, other environmental chemicals may be needed to drive other cancer hallmarks, and genotoxic chemicals will be needed to cause the underlying mutations and genomic instability associated with cancer cells. For these reasons, the obesogenic activity of EDCs may have an even wider impact on disease processes than currently accredited.

One caveat is that the biological availability of the retained compounds in the fatty tissues/cells remains poorly understood. Whilst it is often assumed that xenobiotics retained in fatty deposits of the body would be sequestered, some xenobiotics are known to form fatty acid conjugates which themselves exert local toxicities [93]. Conjugate formation is a metabolic mechanism used to increase hydrophilicity to aid elimination from the body. Common conjugates are with sulphate, glucuronic acid, glutathione or amino acids, and this is also often assumed to negate biological activity. However, for phytoestrogens, whilst sulphation of some does negate oestrogenic activity, sulphation of others can enhance oestrogenic activity [94], and some glucuronide conjugates also retain oestrogenic activity [95]. Although BPA-glucuronide lacks oestrogenic activity, it has been reported as still capable of promoting adipogenesis [96]. The biological availability of pollutants retained in fat is therefore an unresolved aspect which needs further research and specific research to take account not only of enzymatic deconjugation to the free form but also the activity of conjugates themselves.

A further consideration is of the consequence of the timing of exposure to the obesogenic EDCs. Since obesogen exposure in utero or during early postnatal life can increase fat cell number permanently into adult life [4•, 51], it follows that early-life exposure to obesogens could result in a greater lifetime burden of lipophilic pollutants. Whilst the release of lipophilic pollutants in breast milk fat during breastfeeding may serve to detoxify the mother’s breasts, adverse consequences for the baby have long been a concern [97, 98]. However, sudden weight loss, such as through dieting, could also cause the release of lifetime bioaccumulated pollutants from body fat with untoward consequences if the release were too fast for the body to eliminate the compounds sufficiently quickly. A final unanswered question relates to whether pollutant retention may relate to body fat composition as well as quantity of body fat, and therefore whether there may be different consequences arising not just from variations in exposure to EDCs by differing lifestyles but also from variations in the retention of EDCs according to the specific fat composition laid down in the adipocytes.

## Conclusions and Implications for Risk Assessment of Obesogenic EDCs

The weight of evidence pointing towards a role for EDCs in influencing obesity offers not just a mechanistic understanding of the obesity crisis but also a strategy for prevention. Although, undoubtedly, overeating coupled with lack of exercise is a major contributor to the rise in obesity, which can be resolved by reduced calorie intake and increased exercise, it may be that reduction in exposure to obesogenic EDCs, particularly during early life stages, could also contribute to reducing obesity in the population. This will require a political will to limit the use of some of the offending chemicals and an education programme in maternity clinics so that there is a more general understanding of the consequences of exposure to obesogens in early life. Although many of these EDCs were originally developed for a range of beneficial uses, their potential for contributing to obesity needs now to be added into risk assessment of EDCs, and the widespread exposure of the human population to so many EDCs with obesogenic activity requires assessment of the effects not only of individual chemicals but of the complex mixtures entering human tissues from varied lifestyle choices. However, perhaps one of the benefits of this research and its widespread dissemination in the popular media is that informed individuals can also act on a precautionary principle to reduce exposure of themselves and their children ahead of any regulatory actions and therefore have some degree of self-determination in their own exposure to such EDCs.
